# A Review on the Cosmeceutical and External Applications of* Nigella sativa*

**DOI:** 10.1155/2017/7092514

**Published:** 2017-11-22

**Authors:** Ahmad M. Eid, Nagib A. Elmarzugi, Laila M. Abu Ayyash, Maher N. Sawafta, Hadeel I. Daana

**Affiliations:** ^1^Department of Pharmacy, Faculty of Medicine and Health Sciences, An-Najah National University, Nablus, State of Palestine; ^2^Department of Industrial Pharmacy, Faculty of Pharmacy, Tripoli University & National Nanotechnology Project, Biotechnology Research Center, Tripoli, Libya

## Abstract

It is estimated by the World Health Organization (WHO) that most of the world's population depends on herbal medicine for their health care.* Nigella sativa (N. sativa),* also known as black-caraway and as “Kalonji,” is a well-known seed all over the world. It is one of the most common medicinal plants worldwide and contains many useful chemical constituents that we can find in its fixed oil, such as thymoquinone, thymohydroquinone, dithymoquinone, thymol, nigellicine, carvacrol, nigellimine, nigellicine, nigellidine, and alpha-hederin. Due to these numerous important ingredients it was found that it affects different areas of our body and has many pharmacological effects as antibacterial, antiviral, anti-inflammatory, and wound healing effect and also for acne vulgaris, skin cancer, pigmentation, and many cosmeceutical applications. Based on the folklore usage of* N. sativa* seeds and oil, they are used in various systems of food and medicines. The aim of this article is to provide a detailed survey of the literature of cosmeceutical and external applications of* N. sativa* which is expected to stimulate further studies on this subject.

## 1. Introduction

**Figure 1:**
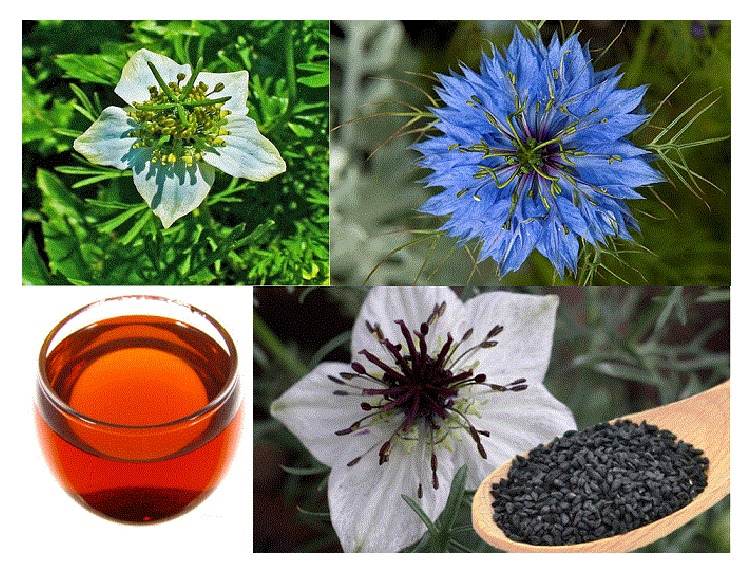
*Nigella sativa *plant, seed, and oil.

*Nigella sativa,* which is a member of the* Ranunculaceae* family [1], can be found all over the world but grows mainly in Eastern Europe, Middle East, and Western Asia [2]. It is a small shrub with tapering green leaves and rosaceous white and purplish flowers. Its fruit contains tiny dark black in color seeds [3] (Figure 1).

The seeds, which have two kinds of oil, fixed and essentials [4], also contain other things such as proteins, alkaloids, and saponins [5, 6]. Much of the biological activity of the seeds has been shown due to the presence of thymoquinone [7], which is the major component of the essential oil but also present in the fixed oil. They have a very low degree of toxicity [8]. Only two patients of contact dermatitis have been reported after topical use. A study in 2003 by Ali and Blunden has shown that taking either the seed extract or its oil will not induce significant toxicity or adverse effects on liver or kidney functions [9]. Therefore, this study proves that* N. sativa* is safe to be used with no significant toxicological or adverse effect.

*N. sativa* is a famous and special plant that has been widely used in different cultures for many centuries [10, 11]. Many studies were interested in this scope such as the seeds of the plant locally known as “Kalonji” which has been used in traditional [12] and alternative medicine for the treatment of a variety of diseases, for example, diarrhea and asthma [13, 14]. Also, many ancient cultures especially in Asia such as Arabian [15] and African countries used black seed oil in various allergies treatment [16]. It was found that the seeds have been used as flavoring agent in food preparation [17]. These magic seeds have been used for treatment of a variety of sicknesses including parasitic diseases [18, 19] and it is used as food preservative as a pharmaceutical powder [20]. Usually developed countries used herbal medicines for the treatment of many diseases such as skin diseases and gastrointestinal diseases such as jaundice, anorexia, dyspepsia, and diabetes [21, 22]. Other uses include hypertension, intrinsic hemorrhage, paralysis, amenorrhea, anorexia, asthma, cough, rheumatism, bronchitis, headache, fever, influenza, eczema, and assorted health care issues [23–25].

The objective of this article is to review the reported cosmeceutical and external application of* N. sativa*. In addition, a review related to the dermatological effects of* N. sativa* seed, its oil, and active ingredients will be conducted.

## 2. *Nigella sativa* External Application

*Nigella sativa* has been used for centuries for the treatment of many skin conditions, for dermatological disorder, and in cosmeceutical formulations [26]. For example, it is used for acne vulgaris, burn, wounds, and injury treatment [26–28], anti-inflammatory for different kinds of skin inflammation [9, 29], and skin pigmentation effect [30, 31].

## 3. Antimicrobial Effect

### 3.1. Antibacterial

New antimicrobial agents are intensively investigated due to pathogenic bacterial infections and microbial resistance, which have become a major health problem worldwide and this led to an increase in the use of medicinal plants [32]. Therefore, many studies discussed antibacterial efficacy of black seeds. Examples include thymoquinone, which is a part of black seeds oil, which is found to have bactericidal activity against most bacteria that was included in the study (MICs values ranged from 8 to 32 *μ*g/ml), especially Gram positive cocci types such as (*Staphylococcus aureus* ATCC 25923 and* Staphylococcus epidermidis* CIP 106510). For* Staphylococcus aureus*, clear inhibition of the growth was found by concentration of 300 mg/ml with distilled water (DW) as control. According to the way that they used for testing, a modified paper disc diffusion method was used. However, there was no effect on* E. coli* and* Enterobacter* bacteria [33]. Black seeds have been found effective against* H. pylori* compared with triple therapy [34]. Black seed extract has also been found to have several multidrug resistant clinical bacterial effects [35]. According to those studies, a clear and undeniable antibacterial effect caused by the* N. sativa*, the inhibition of the bacterial growth, was due to the presence of thymoquinone and melanin. Those findings warrant necessity of further and better investigation of this product.

### 3.2. Antiviral

A few recent studies were found about the antiviral activity of* Nigella sativa* extract, for example, a recent study performed in 2013 obtained significant results about the effect of* N. sativa* oil against hepatitis C virus (HCV). Patients with HCV who cannot receive IFN-*α* were given 450 mg of* N. sativa* oil in capsular dosage form. After 3 months of treatment 3 times daily, a decrease in overall viral count was noted. An increase in antioxidant activity was also found, indicating a reduction in the hemolysis of red blood cells and platelet. Other findings were observed such as reduction in blood glucose levels and in the lower limb edema. [36]. These findings suggest that* N. sativa* administration will decrease viral load in patients with HCV and improve oxidative stress, clinical condition, and glycemic control in diabetic patients.

### 3.3. Antifungal

*Nigella sativa* oil showed antifungal activity against most pathogenic fungi [37].* Candida tropicalis*,* Aspergillus flavus,* and thymoquinone, which is the main composition of the oil, showed antifungal activity against most fungal strains [37, 38]. Its antifungal activity compared with antifungal standard drug Amphotericin B. thymoquinone (Table 1) showed more potent activity against fungal strains than Amphotericin B (Table 2). Data is shown in Tables 1 and 2.

It is also found that thymoquinone has not bad antifungal activity with IC_50_ against* Cryptococcus albidus* which is 20.83 *µ*g/ml,* Candida albicans 23.33 µg/ml*,* Issatchenkia orientalis* 25.33 *µ*g/ml, and* Aspergillus fumigatus* 23.40 *µ*g/ml [39].

In another study of* N. sativa* antifungal effect on dermatophyte fungal strains, it was found that the essential oil extract of thymoquinone has an effective antifungal activity on* T. mentagrophytes, M. canis, and M. gypseum*. Cytotoxicity of* N. sativa* essential oil was included in the study and the results showed that the oil in low concentrations had no significant cytotoxicity in the murine macrophages. However, thymoquinone showed higher cytotoxic effect in comparison with essential oil by the same method of study [40].

In addition, Khosravi et al. (2011) concluded that* C. cyminum, Z. clinopodioides, *and* N. sativa *oils possess antifungal activities to inhibit the growth of* A. fumigatus *and* A. flavus*. The antifungal activity of the oils was evident at the morphological level. Due to the antifungal activity of these oils and their availability as natural volatile products, they might be of use in future studies of antifungal agents [41].

Researchers who studied treatment of fungal infections by using natural products found that* N. sativa* has an enhancing antifungal effect [38, 42, 43]. Another research that depended on micro well dilution assay was conducted against three human pathogenic fungal strains* Aspergillus flavus*,* Aspergillus niger,* and* Candida albicans* [6]. Moreover, in 2013 all the extracts of essential* N. sativa* oil showed effective antifungal activity against* C. albicans*,* C. tropicalis*, and* C. krusei* at MIC values of 16–64 *μ*g/ml [44].

A study was made on Tunisian* N. sativa* fixed oil to test its antibacterial and antifungal activity, and the result shows a validation for the folk use of this oil as an antibacterial and antifungal medicine [45]. Therefore, the existence of various chemical compounds in* N. sativa* and their mechanism of action make it a good candidate as an antifungal agent.

### 3.4. Antiparasitic

In a study conducted in 2014, researchers extracted* Nigella sativa* seeds by methanolic extraction and tested it on* Plasmodium yoelii *infection to see its efficacy. It was found that* N. sativa* extract showed 94%, *P* < 0.05, which showed an excellent suppression compared with chloroquine, which is the drug of choice for* Plasmodium yoelii *infection treatment (methanolic extract of the drug led to 86%). The antimalarial activity was because of antioxidant effect from the extract on* Plasmodium* infected mice. The study was improved to see the antioxidative status in red blood cells, and hepatocytes of infected mice were seen [46, 47].

*N. sativa* oil possesses other activities against cestodes and nematodes action [48]. In a recent study,* N. sativa* oil had an excellent effect in minimizing the total number of* Schistosoma mansoni* worms in liver and reducing the total number of ova that was found in both liver and intestine [23].

Good results were obtained in a study conducted in 2008.* N. sativa* oil and garlic extract were used to see if there are any antischistosomal and antioxidant activity on normal and* Sch. mansoni by *using infected mice. It was noted that the infected mice had an improvement in hematological, biochemical, and antioxidant capacity of schistosomiasis mice compared to the infected untreated ones [49].

In 2002, Aboul-Ela used* N. sativa* oil and* thymoquinone* to test their efficacy against* Sch. mansoni* on infected mice. Results obtained in the study showed decrease in chromosomal abnormalities, especially on chromosomes 2 and 6, and some in chromosomes 13 and 14 when* N. sativa* oil and* thymoquinone* are used in treatment compared with control group [50].

Another study was conducted to determine the effect of* N. sativa* seeds against some parasites such as* Sch. mansoni*,* miracidia*,* cercariae*, and adult worms. Significant results were achieved as a strong effect against all these parasites and even on their eggs was shown. Also* N. sativa* seeds possess an oxidative activity against adult worms which decrease the activities of some enzymes such as glutathione reductase, antioxidant enzymes, and enzymes of glucose metabolism. When these enzymes are damaged, parasite will be weaker and killed [51]. The presence of antioxidant compounds in* N. sativa* may lead to the collection of free radicals and inactivation of them, which may propose significant marketing advantage, due to consumer preference for antioxidant rich products.

## 4. Wound Healing

Thymoquinone is reported to prevent oxidative injury, act as antioxidant, and prevent membrane lipid peroxidation in tissues; these effects suggested the application of* Nigella sativa* topically to accelerate wound healing [27]. A study on wound model rates was done to evaluate the wound healing effect of* N. sativa *oil. The results have shown that it increases the wounding process by unknown mechanism compared to silver sulfadiazine, which may be due to anti-inflammatory and immunomodulatory effects. In the future after further studies, we may use* N. sativa *oil instead of silver sulfadiazine to deal with wound [27].

It was found that* N. sativa* oil has good activity on increasing collagen formation and increasing rate of epithelialization. Thus, it has an excellent effect as wound healing and moisturizing effect [52].

Wound healing process was also observed by Abu-Al-Basal. He saw that when ether extract of* N. sativa* seed was applied on skin, it improved the healing process by decreasing the total and absolute white blood cells count, reducing tissue damage and decreasing bacterial expansion [53].

A study conducted in 2004 used a monolayer prototype of human gingival fibroblast to test wound healing properties of* N. sativa* extract. Increasing in rate of proliferation was observed and closure activity was seen after using the oil in the study [54]. Thus, according to the results of man of those studies and other researches done, it can be said that* N. sativa* might be promising in treatment of wound healing.

## 5. Anti-Inflammatory

### 5.1. Psoriasis

Psoriasis is common skin condition, which is a hyperproliferative, autoimmune skin disorder and can be itchy and painful. An experimental study was undertaken to see the effect of ethanol extract of* Nigella sativa* seeds in treatment of psoriasis. It was found that* N. sativa* increases the epidermal thickness when case study group is compared to control group that used traditional treatment [55].

Another study was made by Ahmed et al. (2014) to compare asiaticoside and the ethanolic extract of* N. sativa* to see the antipsoriatic effect.* N. sativa* oil was applied in two dosage forms, as an ointment and oral dosage form. They had IC_50_ value of 23.9 *μ*g/ml, which is about the IC_50_ value for asiaticoside (20.13 *μ*g/ml). In conclusion,* N. sativa* oil had better effect as antiproliferative activity than the compared treatment [56]. It is concluded based on many researches that* N. sativa* has antipsoriatic effect with the best effect obtained with the combination of ointment and the oral dosage form.

### 5.2. Acne Vulgaris

Acne vulgaris is one of the most prevalent human diseases, which is considered an infectious disease. Many researchers studied the effect of* Nigella sativa* oil against acne vulgaris. Hadi and Ashor (2010) noticed that using 20% of* N. sativa* oil extract in lotion formulation has a better efficacy and is less harmful than benzoyl peroxide lotion 5%, which is the basic treatment for mild to meddle stage of acne vulgaris [57].

A detailed study was conducted on 62 patients. People who have acne and used* N. sativa* lotion as a therapy showed a good decrease in their inflammation and overall number of lesions, the same as benzoyl peroxide. In percentage, more than 50% of patients who used* N. sativa* lotion had good results, but those using benzoyl peroxide lotions showed up to 50% fewer lesions. Of the patients who used* N. sativa* lotion, 20% showed little adverse effects compared to those who used the traditional therapy. However, after patients stopped their scheduled therapy with both lotions, the number of lesions rose 8 weeks after the end of therapy [57].

*N. sativa* seeds have long been used as an external application for different kinds of skin disease. Bhalani and Shah decided to test* N. sativa *oil antibiotic effect compared with standard drug amoxicillin. The results of both treatments were the same in bacterial zone inhibition. In their recent studies they prepared gel dosage form from the oil and Carbopol 940 and tested this formulation as a therapy for acne vulgaris and good results were obtained [58]. Therefore,* N. sativa* is a good candidate in the treatment of inflamed skin which can be caused by infection, irritation, rashes, dermatitis, acne, and psoriasis.

## 6. Skin Pigmentation

### 6.1. Vitiligo

*Nigella sativa* oil as shown before is very effective treatment for different kinds of diseases such as vitiligo, which is a hypopigmentation disorder causing considerable psychological morbidity in a large proportion of its sufferers. Some studies have focused on this point.

A research was made on patients suffering from vitiligo lesions. Researchers used fish oil and* N. sativa* oil as a therapy. Good results were obtained in decrease in the lesions size. It was then decided to include* N. sativa* oil in the basic treatments [31].

A special study was made and noticed that* N. sativa *has the ability to spread melanin within the skin. A possible explanation of the mechanism of this action was that it increased the intensity of melanin by increasing the sensitivity of cholinergic receptors inside the melanopsin, the external part of lizard. That study led them to think about using the active ingredient of* N. sativa oil, *which is thymoquinone, for external problems such as decline in skin pigmentation and vitiligo [30].

## 7. Cosmeceutical Application of* Nigella sativa*

In 2000, it was mentioned that* Nigella sativa* seeds could be used in cosmetics because of its aroma components [59]. After that, it was noticed that sun protective factor (SPF) value for* N. sativa* seed oil is more than 2, so it has some properties against the sun. That means we can use the oil in cosmetics (Table 3) [60].

## 8. Conclusion

In conclusion, this review identified a detailed description on the external application and dermatological application of* Nigella sativa* with a focus on its cosmeceutical application. Historical evidence showed the relation between* N. sativa* and human health care system from decay to modern times, which is due to its antimicrobial, anti-inflammatory, and antifungal activity with application for variety of diseases like bronchitis, cough, asthma, hypertension, paralysis, amenorrhea, anorexia, and rheumatism. In addition, its low degree of toxicity makes it trusted to be used.

## Figures and Tables

**Table 1:** IC_50_ thymoquinone.

	IC value	IC value
*Candida parapsilosis*	IC_50_	2.308 *µ*g/ml
*Cryptococcus laurentii*	IC_50_	9.313 *µ*g/ml

**Table 2:** IC_50_ Amphotericin B.

	IC value	IC value
*Candida parapsilosis*	IC_50_	57.68 *µ*g/ml
*Cryptococcus laurentii*	IC_50_	15.50 *µ*g/ml

**Table 3:** Some commercial products containing *Nigella sativa* extract.

Name of product	Company	Use	Dosage form
Immuno-Viva Core	Immuno-Viva	Natural antioxidant supplement	Capsule and liquid form
Al Barakah	Shiffa Home	Increasing immunity and retaining good health	Soft gelatin capsule
Blackseed Soap	Hemani	Body soap cleaner	Soap
Vatika Black Seed Hair Mask	Vatika Nature	Hair mask	Cream
Vatika Naturals Black Seed Enriched Hair Oil Complete Hair Care	Vatika Nature	Complete hair care, improved shine, texture, and volume, and reduced hair problem	Oil
Vatika Black Seed Shampoo	Vatika Nature	Strong and shiny hair	Shampoo
Black Seed Cream	Hemani	Helping in relaxation	Cream
*Nigella sativa* Cream	Bergmeister	Skin cream	Cream
